# Interpreting Endodontic–Periodontal Lesions: A Conceptual Framework Based on Pulpal and Periodontal Findings—A Narrative Review

**DOI:** 10.3390/dj14030158

**Published:** 2026-03-10

**Authors:** Shungo Komichi, Yusuke Takahashi, Takashi Kawahara, Takayoshi Nagahara, Mikako Hayashi

**Affiliations:** 1 Department of Restorative Dentistry and Endodontology, Graduate School of Dentistry, The University of Osaka, Suita 5650871, Osaka, Japan; 2 Nishinomiya-Kitaguchi LIFE Dental Clinic, Nishinomiya 6638204, Hyogo, Japan; 3 Kawahara Dental Clinic, Takatsuki 5690804, Osaka, Japan; 4 Department of Dentistry, Nippon Kokan Fukuyama Hospital, Fukuyama 7210927, Hiroshima, Japan

**Keywords:** endodontic–periodontal lesions, endo–perio lesions, pulp vitality, vital pulp therapy, treatment timing, clinical framework

## Abstract

**Background/Objectives**: Endodontic–periodontal lesions (EPLs) represent complex pathological conditions arising from interactions between endodontic and periodontal infections. Existing classification systems primarily describe the etiology or periodontal tissue destruction but provide limited support for clinical interpretation, particularly pulp preservation and the sequencing of endodontic and periodontal management. This review aimed to propose a biologically informed conceptual framework intended to organize clinical reasoning considerations in EPL management. **Methods**: This narrative review integrates current knowledge regarding pulp vitality, pathways of infection, periodontal healing, and treatment sequencing reported in the endodontic and periodontal literature. Based on this synthesis, a conceptual framework was constructed using two clinical dimensions: the extent of remaining vital pulp and the presence or absence of coronal-originating infection (IC), defined as a potential coronal pathway of bacterial ingress that may contribute to lesion development. **Results**: The framework categorizes EPLs into four principal groups according to pulp vitality (vital/non-vital) and IC status (present/absent), with additional grading to describe the potential feasibility of pulp preservation and structural reassessment during initial management. Rather than prescribing specific therapies, the model organizes clinical interpretation related to pulp-preserving considerations, the timing of periodontal intervention, and evaluation of surgical management following nonsurgical treatment. **Conclusions**: This framework provides a biologically oriented conceptual model for understanding EPLs and structuring clinical reasoning. It is intended as a hypothesis-guided interpretive framework rather than a clinical practice guideline and is designed to support case interpretation, not to determine mandatory treatment decisions; thus, its clinical applicability requires further validation, and prospective multicenter studies are necessary before routine clinical implementation.

## 1. Introduction

Endodontic–periodontal lesions (EPLs) are complex pathological entities arising from the intricate interplay between endodontic and periodontal diseases, often presenting substantial diagnostic and therapeutic challenges for clinicians [1,2]. Over the decades, several classification systems have been proposed, including etiology-based frameworks—such as Simon’s classification [3]—and the 2018 American Academy of Periodontology (AAP)/European Federation of Periodontology (EFP) classification, which focuses on the extent of periodontal tissue destruction [4,5]. Although these classification systems have substantially improved understanding of EPL pathogenesis and prognosis, they were not primarily designed to determine treatment sequencing. As a result, clinicians may still encounter uncertainty when determining pulp preservation, the timing of subgingival instrumentation, or the selection of surgical approaches. Consequently, in daily clinical practice, diagnostic ambiguity often results in clinicians adopting a pragmatic sequence in which root canal treatment (RCT) is undertaken first, followed by periodontal therapy after an interval of approximately three months, while the biological status of the remaining vital pulp and periodontal ligament may not always be explicitly considered during initial treatment sequencing [6,7]. In many cases, an endodontic-first treatment sequence is adopted as a rational strategy for controlling potential intraradicular infection and reassessing periodontal healing. However, this approach may not fully reflect the biological heterogeneity of EPL presentations. Importantly, this limitation does not diminish their established diagnostic and prognostic value but indicates situations in which additional interpretive guidance may be helpful.

In recent years, the importance of vital pulp therapy (VPT) has been increasingly recognized and supported by both the American Association of Endodontists (AAE) and European Society of Endodontology (ESE) [8,9]. Preservation of pulp vitality in EPLs may have clinical relevance and be biologically desirable in carefully selected cases. Nevertheless, the current classification systems lack explicit diagnostic criteria for assessing the feasibility of pulp preservation and, in many instances, implicitly presuppose RCT as an inevitable intervention. At the same time, vitality-preserving strategies also encompasses potential risks. Misclassification of the pulpal status may result in delayed endodontic treatment, pulpal necrosis progression, or the development of acute symptoms. Therefore, any attempt to preserve pulp vitality in EPLs warrants careful case selection and close clinical monitoring.

Likewise, regarding the timing of subgingival instrumentation, a three-month waiting period following endodontic treatment has traditionally been recommended [6]. However, recent randomized controlled trials have indicated that the optimal timing varies among cases and that excessive delays may increase the risk of periodontal disease progression [10,11,12]. Despite these findings, no structured framework currently exists to guide the timing of periodontal interventions according to specific clinical conditions. Given that such decisions directly influence the treatment duration, invasiveness, and prognosis, a structured approach to support clinical decision-making may be beneficial.

Furthermore, when nonsurgical therapy fails to achieve complete resolution, surgical intervention becomes warranted [13]. The decision between surgical endodontic therapy, periodontal regenerative procedures, or their combined application depends on the underlying pathology; however, this dimension has not been adequately addressed in existing classification systems.

Importantly, these considerations are not intended to promote a specific therapeutic preference but highlight clinical situations in which interpretation of the pulpal–periodontal relationship may be uncertain in clinical assessment. In this context, this review proposes a conceptual framework intended to support clinical interpretation rather than to prescribe mandatory therapeutic protocols. This framework first delineates fundamental clinical groups based on the biological feasibility of pulp preservation and appropriate timing of periodontal intervention. Within each group, further grading is performed to stratify pulp-preserving approaches according to the extent of the remaining vital pulp and to guide the selection of surgical strategies based on the response to nonsurgical management. By integrating biological considerations with stepwise interpretation of clinical findings, this framework aims to organize clinical reasoning in situations where the biological relationship between pulpal and periodontal components is uncertain and may thereby facilitate more individualized and biologically oriented management. This framework is intended as a hypothesis-generating perspective and warrants prospective clinical validation.

## 2. Existing Classifications and Their Limitations

The following discussion is not intended to invalidate existing classification systems, which remain essential for diagnosis, communication, and prognostic assessment. Rather, the limitations described below relate specifically to their role in guiding treatment sequencing and clinical decision-making in EPL cases where the pulpal–periodontal relationship is uncertain.

### 2.1. Classification Based on Pathogenesis

The interrelationship between endodontic and periodontal diseases has long been recognized, and several classification systems have been proposed to describe their pathogenesis [3,14,15,16,17]. Among these, Simon’s (1972) classification remains the most influential, categorizing EPLs into five groups according to their route of infection [3]:

1. Primary endodontic lesions;
2. Primary endodontic lesions with secondary periodontic involvement;
3. Primary periodontic lesions;
4. Primary periodontic lesions with secondary endodontic involvement;
5. True combined lesions.

The primary objective of Simon’s framework is to identify the infection pathway and determine the most appropriate treatment strategy. This concept subsequently influenced the classifications of Torabinejad and Trope (1996), based on the origin of periodontal pockets [18], and the 1999 AAP World Workshop, which categorized lesions as endodontic–periodontal, periodontal–endodontic, or combined [19]. Al-Fouzan (2014) further refined the model by emphasizing the distinction between primary and secondary involvement [20].

These pathogenesis-based classifications have contributed substantially to a systematic understanding of EPL development and remain widely referenced in both clinical practice and undergraduate education. They are particularly useful for early-stage lesions, where identifying the primary etiologic source helps prevent unnecessary intervention.

However, their clinical applicability is limited. A definitive preoperative classification is often unfeasible because diagnostic clarity typically emerges only after treatment initiation. In cases with unclear treatment histories or previous RCT, a definitive diagnosis is often difficult. For example, in Simon’s classification, it is sometimes impossible to distinguish between primary endodontic lesions with secondary periodontic involvement and true combined lesions. Given these diagnostic challenges, such classifications may not always distinguish cases in which pulp vitality could potentially be preserved during initial treatment planning.

### 2.2. Classification Based on the Destruction of Teeth and Periodontal Tissues

At the 2018 World Workshop, AAP and EFP proposed the following new framework, emphasizing the presence or absence of root damage and extent of periodontal tissue destruction [4,5]:

1. EPLs with root damage:
  - Root fracture or cracking;
  - Root canal or pulp chamber perforation;
  - External root resorption.
2. EPLs without root damage:
  - In patients with periodontitis (Grades 1–3, based on pocket depth and extent);
  - In patients without periodontitis (Grades 1–3, same criteria).

This system facilitates diagnosis and aids in estimating prognosis, helping clinicians determine the general treatment direction, particularly whether to preserve or extract the affected tooth.

Nevertheless, this framework assumes that both endodontic and periodontal therapies are required for nonsurgical management [4,21]. Consequently, the system may not always identify situations in which pulp vitality could potentially be preserved, which limits its usefulness when considering minimally invasive or vitality-preserving strategies.

### 2.3. Variability of Clinical Decision-Making in EPL Management

Although previous classifications systematized EPLs from specific perspectives, they remain inadequate for directly guiding clinical management. In daily practice, a standardized protocol—“perform RCT first and reassess periodontal intervention after three months”—is frequently applied. However, therapeutic strategies for EPLs should be more diverse and individualized, encompassing the following three key considerations.

#### 2.3.1. Possibility of Pulp Preservation

In patients with EPLs, root canal infections may spread through the apical foramen [22,23], lateral canals [24,25], or exposed dentinal tubules [26,27], leading to inflammation and impaired healing of periodontal tissues. Accordingly, when an intracanal infection is evident in these patients, an endodontic-first approach is essential to control the infection.

By contrast, endodontic intervention may be unnecessary for EPLs without an intracanal infection, as exemplified in cases of primary periodontal lesions in which the pulp has been described by the authors as “vital healthy pulp” that does not require extirpation [3]. Nevertheless, prophylactic RCT is often performed to prevent secondary necrosis resulting from vascular injury during periodontal surgery. Conversely, case reports have revealed that, with careful management, healing may occur through periodontal therapy alone without pulpal intervention [28].

Moreover, even in patients with EPLs along with intracanal infection, pulp-preserving strategies may be considered when viable pulp tissue remains at the canal orifices in one or more roots. This situation is frequently encountered in multirooted teeth affected by EPLs, where partial pulp necrosis may be present in some roots while viable pulp tissue is preserved in others. In such cases, treatment may involve root-specific partial pulp preservation, in which infection in affected roots is selectively eliminated by RCT, while viable radicular pulp is preserved through pulpotomy at the level of the canal orifices in selected roots. This approach aims to maintain the physiological function of the remaining pulp tissue while achieving adequate infection control. Recent studies have demonstrated the feasibility of such vital pulp therapy–based strategies [29,30].

From a clinical decision-making perspective, the primary consideration in patients with EPLs is whether any vital pulp tissue remains. When no remaining vital pulp is present, RCT is indispensable. By contrast, when vital pulp tissue is preserved, pulp-preserving strategies may be biologically justifiable and should be considered, rather than assuming the inevitably of RCT as the primary intervention.

Accordingly, the pulpal condition in EPLs may be interpreted in two broad categories based on the presence of remaining vital pulp tissue:

1. Absence of remaining vital pulp;

2. Presence of remaining vital pulp to a variable extent.

Further characterization of cases with remaining vital pulp, based on the anatomical distribution and amount of vital pulp tissue, may assist interpretation of the biological status of the tooth rather than selection of specific pulp-preserving procedures.

#### 2.3.2. Timing of Subgingival Instrumentation

When managing EPLs, both the presence of periodontitis and risk of secondary periodontal progression must be considered. Although supragingival debridement is universally beneficial from the initial phase onward, the timing of subgingival instrumentation requires more careful consideration.

In apical periodontitis of endodontic origin, periodontal healing may occur following adequate control of intraradicular infection [31]. In EPLs, periodontal tissues within the pocket may respond favorably following endodontic infection control. If subgingival instrumentation is performed immediately, before periodontal stabilization occurs, this tissue may be disrupted, which may compromise healing [32]. Accordingly, in selected cases, it may be biologically advantageous to complete endodontic treatment first, allow a healing interval to permit periodontal stabilization, and subsequently re-evaluate the need for initiating subgingival instrumentation, rather than adopting an immediate periodontal intervention approach.

Reported recommendations for the interval between endodontic and periodontal therapy range from one to six months, with a two-to-three-month waiting period appearing to represent the most widely accepted clinical consensus [6,7,33,34,35]. Nevertheless, few randomized clinical trials have directly compared delayed and immediate periodontal intervention following endodontic treatment. Notably, several studies have reported comparable or even more favorable outcomes when subgingival instrumentation was performed immediately, without a waiting interval [10,11,12]. However, these studies predominantly involved lesions associated with marginal periodontitis, in which periodontal breakdown was primarily associated with plaque-induced periodontal disease and periodontal improvement following endodontic treatment was unlikely. In such contexts, the biological rationale for delaying periodontal intervention is minimal, and immediate subgingival instrumentation may be justified.

Accordingly, consideration of subgingival instrumentation requires interpretation of whether periodontal findings may be influenced by possible endodontic contribution to the initial lesion formation. Traditionally, EPLs have been categorized into primary endodontic, primary periodontal, and true combined lesions based on their presumed etiologic origin [3,18,19,20]. For lesions with a predominant endodontic component—such as primary endodontic lesions and true combined lesions—in which endodontic pathology may have contributed, at least in part, to lesion and pocket formation, periodontal findings may be difficult to interpret until initial periodontal stabilization occurs following endodontic infection control. By contrast, primary periodontal lesions lack an endodontic contribution, and periodontal findings are more likely to be interpretable without such delay, as the biological rationale for delay is limited.

In summary, distinguishing lesions in which periodontal findings may be influenced by endodontic inflammation from those in which they are not is important for interpreting periodontal status. Subgingival instrumentation is therefore discussed in relation to the interpretability of periodontal findings rather than as a prescriptive timing of intervention.

#### 2.3.3. Selection of Surgical Approaches

When nonsurgical management fails to achieve complete healing, surgical intervention is indicated. Persistent lesions may arise from refractory apical periodontitis, marginal periodontitis, or a combination of both. When the underlying etiology can be inferred from the clinical history or treatment response, treatment planning is relatively straightforward: surgical endodontics for refractory apical periodontitis and regenerative periodontal therapy for marginal periodontitis. However, in many cases, the etiology remains indeterminate, rendering clinical decision-making more complex.

In such diagnostically ambiguous cases, applying an “endodontic-surgery-first” principle can be a rational approach for achieving infection control, as periodontal findings may improve following adequate endodontic treatment, thereby allowing staged clinical decision-making based on treatment response [36,37,38,39]. However, when marginal periodontitis is concurrently present, adjunctive periodontal regenerative therapy becomes necessary, potentially prolonging treatment and increasing invasiveness. Furthermore, gingival recession following endodontic surgery may compromise subsequent regenerative procedures, highlighting the importance of prudent case selection.

Alternatively, several studies have demonstrated favorable outcomes with a simultaneous surgical approach that integrates endodontic surgery and periodontal regenerative therapy within a single session, particularly in diagnostically ambiguous cases [40,41,42,43]. This strategy enables the concurrent management of both potential etiologies, potentially shortening the overall treatment duration and reducing surgical frequency; however, it entails greater procedural complexity, cost, and technical demand.

Therefore, management of persistent or non-resolving EPLs, including staged or simultaneous surgical approaches, depends on the interpretation of the remaining pathology. These considerations include whether the residual findings are compatible with a predominantly periodontal condition or represent an indeterminate situation in which apical and marginal components cannot be clearly distinguished. In such ambiguous situations, clinical response may be organized either in a stepwise manner or in a combined manner, as well as diagnostic accuracy, healing potential, invasiveness, cost-effectiveness, and patient preference.

In summary, clinical assessment of EPLs frequently involves three interrelated perspectives:

1. Interpretation of the pulpal condition;
2. Interpretation of periodontal findings in the context of potential endodontic influence;
3. Interpretation of persistent lesions when healing remains limited after initial therapy.

Nevertheless, these elements have not been sufficiently incorporated into existing classification systems, nor systematically related to underlying biological findings. Accordingly, the present review proposes a conceptual framework intended to facilitate biological interpretation of EPL presentations and organize clinical reasoning during case reassessment, rather than to prescribe specific therapeutic management.

## 3. Proposed Conceptual Framework

### 3.1. Basic Concept

The proposed framework is intended to support structured clinical interpretation of EPLs prior to therapeutic intervention. It applies to teeth demonstrating a continuous radiolucency extending from the marginal to the apical region on radiographic examination, accompanied by a clinical attachment loss reaching the apex—findings considered consistent with EPLs. These lesions are considered in the absence of acute pulpal symptoms and without evidence of root structural damage.

Within this framework, clinical interpretation is organized using two primary clinical parameters:

1. Pulp vitality;

2. The presence or absence of coronal-originating infection (IC).

1. Pulp Vitality (evaluated by EPT)

Conceptually, pulp vitality can be regarded as a continuum reflecting the extent of remaining vital pulp tissue along the root canal, ranging from complete vitality to complete necrosis. For clinical interpretation of the pulpal condition, however, this framework considers pulp vitality in two initial categories (vital vs. non-vital) as indicated by electric pulp testing (EPT), followed by a more detailed evaluation of the extent of remaining vital pulp to assist interpretation during subsequent clinical assessment and reassessment when pulp vitality is detected in the first step.

- Vital (V): A positive response to EPT indicates the presence of at least partially vital pulp tissue. In such cases, evaluation of the extent of remaining vital pulp informs consideration of the biological feasibility of pulp preservation, which may range from observation and pulp-preserving approaches to situations in which RCT may ultimately be required.
- Non-vital (NV): A lack of EPT response is interpreted as findings consistent with pulpal necrosis, a condition in which the biological potential for pulp preservation is absent and management commonly involves endodontic treatment, including RCT.

Pulp sensibility testing was not interpreted as a single definitive diagnostic test. EPT was employed as an initial screening indicator and was interpreted in conjunction with other clinical findings. In multirooted teeth, responses were evaluated at the root level rather than the tooth level because a positive response may originate from a single remaining vital root while the other roots may be necrotic. Therefore, a positive EPT response alone was not considered sufficient evidence of overall pulp vitality. In cases of inconsistent findings or when clinical suspicion remained in multirooted teeth, direct inspection under microscopic magnification using an operating microscope was performed to confirm pulpal status. This stepwise protocol is intended to minimize misclassification caused by false-positive EPT responses.

2. Coronal-Originating Infection

In this review, “coronal-originating infection” (IC) is introduced as an interpretive concept used to consider the relationship between periodontal tissues and endodontic inflammation, suggesting that periodontal pockets may be partially influenced by endodontic inflammation. In such situations, probing depth and attachment loss may not reflect the true periodontal condition while endodontic inflammation persists. IC therefore serves to support interpretation and reassessment of periodontal findings rather than to identify the microbiologic origin of infection.

- IC^+^: The presence of findings suggesting infections originating from the coronal portion—such as deep caries, defective restorations, or cracks that allow bacterial ingress into the pulp chamber—may indicate a potential endodontic contribution to lesion development. In such situations, periodontal findings including pockets may be influenced by endodontic inflammation and may change following resolution of the endodontic component, rendering early periodontal interpretation potentially unreliable. Within this framework, IC^+^ is interpreted only when structural findings suggesting a coronal pathway of bacterial ingress are accompanied by pulpal findings clinically compatible with coronal contamination.
- IC^−^: When IC is not suspected, the lesion is less likely to reflect a coronal endodontic contribution, and periodontal findings are unlikely to be substantially modified by endodontic inflammation. Consequently, interpretation of the periodontal condition does not depend on resolution of the endodontic component and can be considered independently.

Teeth with previous root canal treatment should not be automatically categorized as IC^+^. In previously treated teeth, IC is interpreted as a clinical indicator of suspected persistent or recurrent intraradicular infection (i.e., potential endodontic contribution), rather than being determined by the mere history of endodontic therapy. In this context, “IC” does not imply certainty regarding the original route of infection but serves as an interpretive indicator of endodontic contribution relevant to treatment sequencing. Accordingly, previously treated teeth may be classified as IC^+^ when findings suggest persistent or recurrent endodontic pathology and/or a plausible pathway for reinfection, such as coronal seal breakdown, recurrent caries, clinical symptoms, a sinus tract, progressive or localized periapical radiolucency suggestive of endodontic origin, or radiographic/procedural evidence of inadequate prior root canal treatment (e.g., separated instruments or poorly condensed root canal filling). In the absence of such findings, the case should be managed as IC^−^ or indeterminate with careful follow-up and reassessment.

The framework is intended as a hypothesis-generating model rather than a validated diagnostic protocol. Within this framework, IC should be understood as a clinical interpretive construct indicating that a coronal pathway of bacterial ingress may contribute to lesion development. It is not intended to establish the definitive origin of infection but support clinical interpretation and reassessment when considering the relationship between pulpal and periodontal components and to help organize considerations regarding the timing of periodontal intervention. It should not be interpreted as indicating the histologic status of periodontal tissues or the presence of residual viable periodontal ligament. Because clinical findings may be inconclusive, classification may remain indeterminate and subject to reassessment as additional information becomes available over time. Microscopic assessment is not considered a prerequisite for applying the framework but may serve as an adjunctive method for improving diagnostic confidence.

### 3.2. Combination of V/IC Parameters

By integrating these two diagnostic parameters into a 2 × 2 matrix, four fundamental clinical groups can be conceptualized, providing a structured framework for organizing clinical interpretation prior to therapeutic decision-making. The following group descriptions are intended to characterize patterns of biological behavior and diagnostic interpretability. Any reference to specific treatment approaches is provided solely as an example of how different biological scenarios may influence clinical reasoning and should not be regarded as mandatory management.

1. V/IC^+^: Vital pulp with IC.

The presence of a vital pulp despite IC may represent a biological condition in which partial pulp preservation (pulpotomy) is possible. Periodontal findings in such cases may change following resolution of the endodontic inflammatory component, and their diagnostic interpretation may therefore require reassessment after an interval of healing.

2. NV/IC^+^: Non-vital pulp with IC.

In these cases, the biological potential for pulp preservation is limited rather than entirely absent, and management often involves RCT. Because endodontic inflammation may also contribute to the periodontal presentation, periodontal findings may not reliably represent the definitive periodontal condition until after resolution of the endodontic inflammatory component and an interval of healing.

3. V/IC^−^: Vital pulp without IC.

These cases, characterized by the absence of IC, may represent a biological condition compatible with preserving pulpal vitality, including partial pulp preservation via pulpotomy and, where feasible, conservative management without pulpal intervention. Even when endodontic treatment becomes necessary, periodontal findings are unlikely to change solely following resolution of the endodontic component. Consequently, evaluation of the periodontal condition need not depend on endodontic healing, and reliable periodontal assessment does not require a prior observation period for resolution of endodontic inflammation.

4. NV/IC^−^: Non-vital pulp without IC.

In such cases, the absence of vital pulp indicates that pulp preservation is not feasible, and the periodontal findings are unlikely to change solely with resolution of endodontic inflammation. The periodontal condition may therefore be interpreted independently of endodontic healing, as additional healing of the endodontic component is unlikely to alter the diagnostic interpretation of the periodontal findings.

The prognosis of EPLs is closely associated with the extent of periodontal tissue destruction [23,44].

For cases with vital pulp (V/IC^+^ and V/IC^−^), grades V_1_–V_3_ are proposed according to the extent of remaining vital pulp.

**Figure 1:**
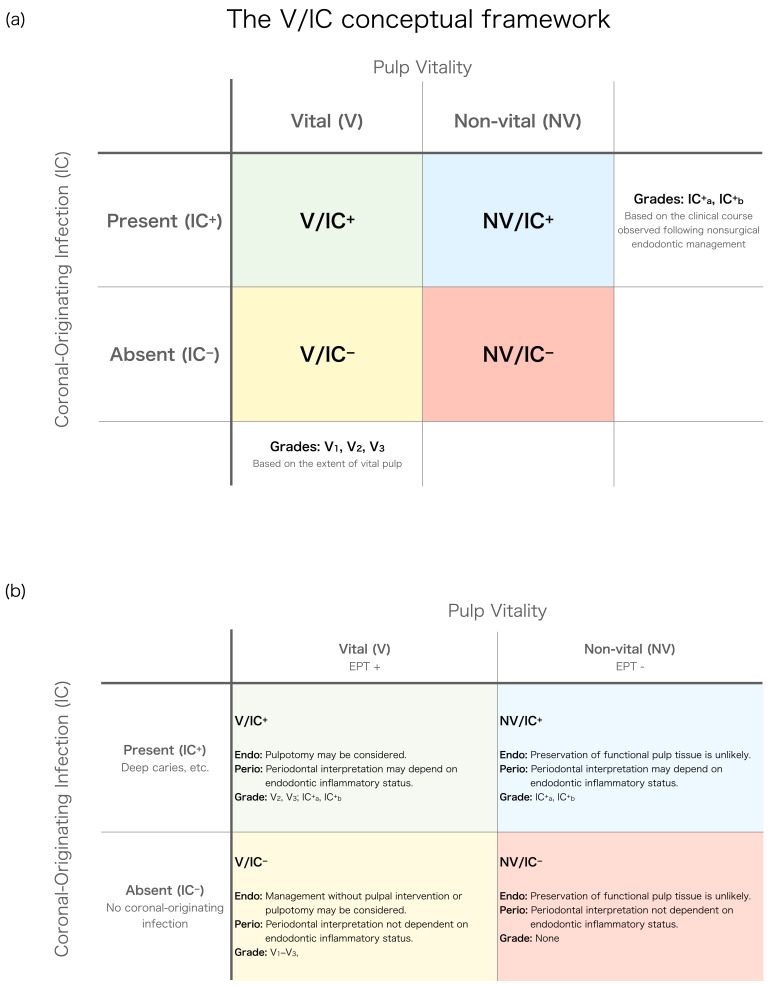
The V/IC conceptual framework for interpreting endodontic–periodontal lesions (EPLs). (**a**) Primary diagnostic groups based on pulp vitality (assessed by electric pulp testing, EPT) and the presence or absence of coronal-originating infection (IC), defined as infections originating from the coronal portion that may contribute to EPL development. The four groups (V/IC^+^, NV/IC^+^, V/IC^−^, NV/IC^−^) represent combinations of pulp vitality and IC status. Grades V_1_–V_3_ represent the extent of remaining vital pulp, while IC^+^_a_ and IC^+^_b_ indicate potential persistent etiologic patterns inferred from the clinical course following nonsurgical endodontic management and assist interpretation of underlying pathology. (**b**) Illustrative clinical implications corresponding to each diagnostic group. V = vital pulp; NV = non-vital pulp; IC^+^ = IC present; IC^−^ = IC absent.

When nonsurgical measures do not result in resolution, the subsequent clinical course may provide additional information regarding the underlying pathology. Beyond its role in the primary classification, IC also functions as a parameter in secondary interpretation, particularly in cases showing persistent findings after initial endodontic management. In IC^−^ cases, the lesion is more consistent with a predominantly periodontal process, whereas in IC^+^ cases persistent findings may reflect marginal periodontitis, refractory apical periodontitis, or a combination of both. Accordingly, grades IC^+^_a_ and IC^+^_b_ are defined to describe potential persistent etiologic patterns following endodontic management and assist interpretation of these lesions (Figure 1a,b).

### 3.3. Grading of Residual Pulp Vitality (V Group)

Consistent with the stepwise interpretive framework described in Section 3.1, grading within the V group focuses on a detailed assessment of residual pulp vitality in teeth initially classified as vital based on EPT. This grading system describes the extent of remaining vital pulp tissue along the root canal in EPLs. Accurate recognition of residual pulp vitality is important for understanding the biological status of the tooth, as misinterpretation may affect subsequent endodontic and periodontal assessment. Aravind et al. reported that 94.7% of teeth exhibited a positive response to EPT after full pulpotomy, whereas only 13.4% responded to cold testing [45]. These findings indicate that EPT exhibits superior sensitivity in detecting residual radicular pulp vitality, while cold testing carries a higher risk of false-negative results when assessing indications for pulpotomy. Accordingly, EPT serves as the principal clinical reference in this framework. Cold testing and intraoperative microscopic examination of the pulp are used as complementary assessments to support interpretation of pulpal status. Importantly, final V grading (particularly V_2_ and V_3_) is not based solely on preoperative pulp sensibility findings but is interpreted in light of intraoperative observations, including pulpal bleeding and root-specific pulp condition under an operating microscope.

The V classification is intended as a provisional clinical categorization requiring clinical monitoring and reassessment rather than a definitive biological diagnosis. For cases interpreted as V_1_, in which no pulpal intervention is performed, the categorization remains provisional and requires confirmation through longitudinal follow-up and reassessment of clinical and radiographic findings. In cases where the distinction between V_2_ and V_3_ is uncertain, continued observation with follow-up reassessment may be appropriate before definitive clinical intervention is considered.

**Figure 2:**
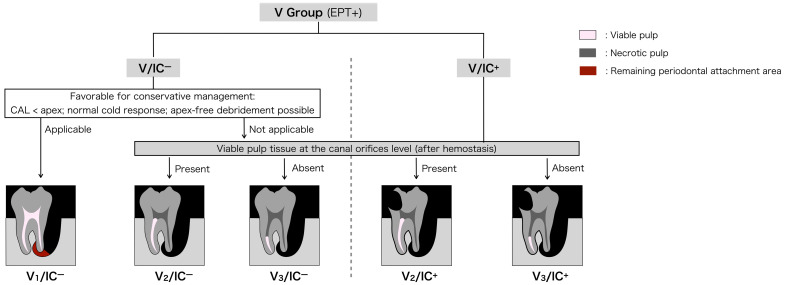
Conceptual illustration of clinical interpretation for EPLs with vital pulp (V group, EPT+). The diagram schematically depicts how clinical findings may be interpreted in relation to pulp-preserving considerations and possible endodontic involvement. The figure is intended to support understanding of the proposed framework and does not represent a treatment algorithm or prescribe specific management decisions. Cases with vital pulp are first interpreted according to the presence (V/IC^+^) or absence (V/IC^−^) of IC. In V/IC^−^ cases, a clinical presentation consistent with conservative management without pulpal intervention (Grade V_1_/IC^−^) may be interpreted if the CAL does not reach the apex, cold testing elicits a normal response, and periodontal debridement can be performed without contacting the apical foramen. If these conditions are not supportive of a V_1_ interpretation, endodontic access may be performed as part of clinical management, and further grading is interpreted according to the presence (Grade V_2_) or absence (Grade V_3_) of viable pulp tissue at the canal orifice level, as assessed by direct microscopic observation after achieving hemostasis. V/IC^+^ cases are similarly interpreted into grades V_2_ and V_3_ based on the extent of remaining viable pulp. Color codes: pink = viable pulp; dark gray = necrotic pulp; red = remaining periodontal attachment area. EPLs = endodontic–periodontal lesions; IC = coronal-originating infection; CAL = clinical attachment level.

For teeth with vital pulp (V/IC^+^ and V/IC^−^), three grades (V_1_–V_3_) are established based on the extent of remaining vital pulp (Table 1, Figure 2).

1. Grade 1 (V_1_): Clinically healthy pulp

This grade applies exclusively to V/IC^−^ cases in which no IC is detected. V_1_ is assigned when three conditions are fulfilled:

- The clinical attachment level (CAL) does not extend to the apical foramen;
- The pulp exhibits a normal response to cold testing;
- Periodontal debridement can be performed without contacting the apical foramen.

Even if radiographs show periapical radiolucency surrounding the apex, the presence of remaining periodontal attachment adjacent to the apical foramen (red area in Figure 2) suggests that pulpal infection has not yet occurred. Under these conditions, the pulp is interpreted as clinically healthy based on favorable diagnostic findings, and pulpal intervention is not biologically indicated at the time of assessment. Because clinical assessment of pulpal status is subject to uncertainty, the initial classification should be regarded as provisional. Subsequent clinical findings over time may lead to reassessment and reclassification as the biological condition evolves.

2. Grade 2 (V_2_): Vital pulp present at the canal orifices

This grade applies to cases in which after removing infected coronal pulp and achieving hemostasis, viable pulp tissue is confirmed at the canal orifice level under microscopic observation. This pattern reflects a heterogeneous pulpal status among roots, which is frequently encountered in multirooted teeth affected by EPLs. The findings indicate that a portion of radicular pulp tissue retains biological viability, whereas infection is confined to other regions. Accordingly, Root-specific partial pulp preservation may be biologically feasible in selected roots while infection control is required in others.

3. Grade 3 (V_3_): Vital pulp confined to apical third

This grade applies to cases in which a positive response to EPT suggests residual pulpal vitality, but no viable pulp tissue is observed at the canal orifices level in any root after removal of infected coronal pulp and achievement of hemostasis. Accordingly, the remaining vital pulp is presumed to be confined to the apical third of the root canal system. Under these conditions, preservation of functional radicular pulp is unlikely, and RCT may be considered a practical management approach for controlling intracanal infection and enabling subsequent clinical reassessment. Although limited vital tissue may remain in the apical third, reliable clinical methods for confirming sterility and long-term functional preservation are currently lacking.

In V/IC^+^ cases, access to the pulp chamber is required, and grade differentiation between V_2_ and V_3_ is assessed through direct microscopic evaluation of the pulp (Figure 2).

By contrast, V/IC^−^ cases may include grade V_1_. Identification of V_1_ requires interpretation of findings consistent with clinically healthy, vital pulp without endodontic intervention, which is particularly challenging in multirooted teeth. Because EPT may yield a positive response even when partial pulpal necrosis is present, interpretation relies on combined assessment of both the tooth’s response to cold testing and condition of the periodontal attachment adjacent to the root apex. If clinical and diagnostic findings do not sufficiently support interpretation as V_1_, the possibility of partial pulpal necrosis should be considered, and endodontic access may be warranted for therapeutic purposes. Furthermore, even in the absence of clear diagnostic signs of pulpal necrosis, endodontic intervention may be considered when periodontal debridement extending to the apical region cannot be performed without contacting the root apex. In such situations, pulp therapy is undertaken not due to suspected necrosis, but as a procedural necessity to facilitate thorough debridement. During endodontic access, direct microscopic evaluation of the pulp allows definitive differentiation between grades V_2_ and V_3_ (Figure 2).

In selected vital teeth with deep structural compromise (e.g., deep caries, defective restorations approaching the pulp, or cracks extending toward the pulp chamber), differentiation between IC^+^ and IC^−^ based on noninvasive findings alone may be unclear. In such borderline cases, diagnostic pulp exposure may be used as a confirmatory step for clarifying the clinical pulp condition.

If the exposed pulp appears clinically intact with normal bleeding characteristics, the lesion is interpreted as lacking a coronal endodontic contribution (IC^−^), and pulp-preserving management with concurrent periodontal therapy may be undertaken. Conversely, when partial pulpal necrosis or contaminated pulp tissue is identified, the etiologic origin of pulpal breakdown cannot be accurately determined, as secondary pulpal necrosis may also occur in primarily periodontal lesions. For clinical management, such cases may be treated as IC^+^ as a precautionary clinical interpretation to prioritize control of a possible endodontic contribution to initial lesion formation, followed by periodontal reassessment after initial infection control.

Because bacterial invasion through dentinal tubules or microcracks may not be clinically evident, IC may occasionally be underestimated in teeth that retain pulp sensibility. This uncertainty is clinically relevant primarily in vital teeth in which pulp preservation is being considered; therefore, careful follow-up reassessment is an integral component of the framework rather than an exception. In teeth lacking pulp sensibility, endodontic management is already indicated, and potential misclassification of IC has less influence on the initial management sequencing.

### 3.4. IC and Interpretation of Periodontal Findings During Reassessment

For interpreting periodontal findings during reassessment after endodontic treatment, particularly when considering subgingival instrumentation, it is first necessary to consider whether periodontal healing may occur following endodontic infection control. This is essentially equivalent to assessing whether endodontic factors contribute, at least in part, to lesion development. Various diagnostic indicators—such as responses to EPT and radiographic characteristics of the lesion—have traditionally been used to determine the presence of endodontic contribution. However, one clinically useful interpretive indicator is IC.

Although IC may be suspected radiographically, a definitive diagnosis requires direct visual inspection after the removal of caries or defective restorations. If infection extends into the pulp chamber and any degree of pulpal necrosis is present, the case may be categorized as IC^+^ within this clinical framework. Because IC spreading through cracks can be subtle or clinically undetectable, examination under a dental operating microscope may provide additional interpretive information.

Therefore, identification of IC may assist interpretation of periodontal findings during reassessment, rather than determining a specific treatment timing. However, interpretation can be challenging in certain clinical situations—for example, when bacterial invasion occurs via dentinal tubules without obvious coronal pulp exposure—making the distinction between IC^+^ and IC^−^ less straightforward. Moreover, even in IC^+^ cases, clinical periodontal response can vary widely—from minimal periodontal involvement to advanced periodontal destruction in long-standing lesions. Accordingly, longitudinal evaluation of CAL changes during follow-up after endodontic therapy provides context for understanding periodontal healing and distinguishing residual endodontic resolution from true periodontal defects.

In IC^+^ cases, non-resolving periodontal pockets may suggest persistent endodontic pathology as a contributing factor. Accordingly, a grading system is proposed to support clinical interpretation during reassessment.

### 3.5. Interpretive Grades in IC^+^ Presentations

The time intervals described in this section are not intended to prescribe the timing of periodontal or surgical intervention. Rather, they represent approximate observational periods during which the influence of endodontic inflammation may diminish and lesion behavior may become distinguishable, thereby assisting interpretation of the underlying pathology.

**Figure 3:**
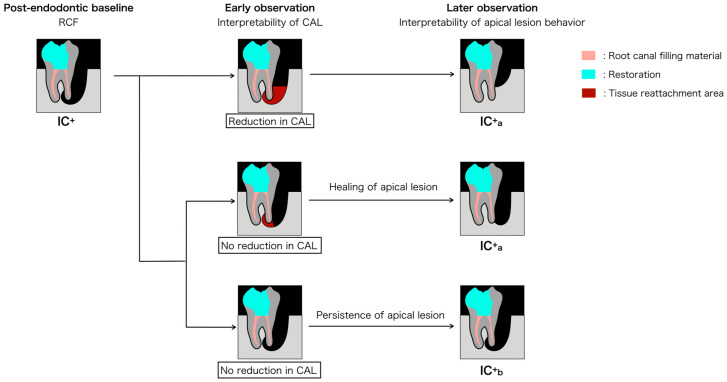
Interpretive framework for IC^+^ cases. After completion of root canal filling (RCF; baseline), periodontal findings are re-evaluated during early observation, followed by later radiographic or cone-beam computed tomography (CBCT) observation of apical lesion behavior. Grade a (IC^+^_a_): A reduction in CAL is interpreted as a clinical response compatible with resolution of endodontic contribution to initial lesion formation. Alternatively, unchanged CAL with radiographic evidence of apical healing is also interpreted as IC^+^_a_. These findings suggest a reduced likelihood of persistent endodontic influence. Grade b (IC^+^_b_): Unchanged CAL without radiographic evidence of apical healing is interpreted as persistent or uncertain endodontic contribution. These findings indicate continued ambiguity in the pulpal–periodontal relationship and support continued reassessment. Color codes: pink = root canal filling material; light blue = restoration; red = tissue reattachment area. IC = coronal-originating infection; CAL = clinical attachment level.

In IC^+^ presentations, persistent periodontal pockets after endodontic treatment may indicate residual pathology originating from refractory apical periodontitis, marginal periodontitis, or both. To assist interpretation of these complex presentations, the grade framework considers the healing tendency of apical lesions—assessed during an early observation phase, in which CAL becomes interpretable, and a later observation phase, in which the behavior of the apical lesion becomes interpretable, using radiographic or cone-beam computed tomography (CBCT) observation [22,46]. CBCT findings are considered adjunctive information and are not a prerequisite for application of the framework. In this framework, IC^+^_a_ and IC^+^_b_ are differentiated according to the clinical course following nonsurgical endodontic treatment, supporting continued clinical reassessment and refinement of interpretation (Figure 3). Surgical procedures are discussed only as possible clinical contexts encountered after reassessment when periodontal healing remains limited.

#### 3.5.1. Grade a (IC^+^_a_): Healing Tendency of Apical Periodontitis

A lesion is classified as IC^+^_a_ when reduction in CAL is observed or CAL remains unchanged but radiographic or CBCT imaging demonstrates apical healing. In such cases, the endodontic pathology is interpreted as showing a healing tendency, and the periodontal findings may be considered in the context of diminishing endodontic influence. Because maturation of the apical attachment may require time, interpretation of stable hard tissue repair typically becomes appreciable only during later observation. At this stage, interpretation of periodontal findings becomes more reliable, as the stability of the apical attachment can be distinguished from transient healing changes.

**Figure 4:**
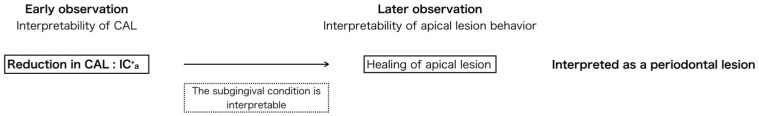
Lesions showing CAL reduction during early observation classified as IC^+^_a_. At this stage, the subgingival condition becomes interpretable, whereas assessment of suitability for periodontal regenerative therapy is deferred until apical healing is confirmed by radiographic observation during later observation. These diagrams depict interpretive scenarios and do not represent a mandatory treatment protocol. CAL = clinical attachment level.

**Figure 5:**
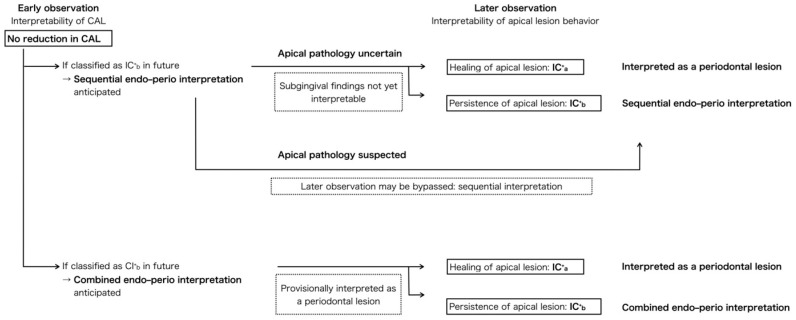
Reassessment framework when no reduction in CAL is observed at the three-month re-evaluation. When an IC^+^_b_ presentation is considered possible but not yet confirmed pending lesion morphology reassessment, findings at early observation may be interpreted within alternative clinical contexts corresponding to sequential or combined endo–perio interpretation. At later observation, lesion morphology is further interpreted to support subclassification (IC^+^_a_ or IC^+^_b_) and provide context for understanding subsequent clinical management considerations. In cases with suspected apical pathology, interval reassessment may be shortened and earlier re-evaluation of the pulpal–periodontal condition may be appropriate without waiting for complete lesion resolution. The term "apical pathology uncertain" refers to situations in which apical involvement cannot be determined based on current clinical and radiographic findings, whereas "apical pathology suspected" indicates findings compatible with refractory apical pathology. These diagrams depict interpretive scenarios and do not represent mandatory treatment protocols. IC = coronal-originating infection; CAL = clinical attachment level.

If CAL reduction is evident during early reassessment, the subgingival periodontal findings become more interpretable and are less likely to be influenced by residual endodontic inflammation. Figure 4 illustrates a possible clinical interpretation of this lesion behavior pattern and is not intended to prescribe a specific treatment sequence. Rather, it depicts how understanding of the pulpal–periodontal relationship may evolve as additional clinical information becomes available over time (Figure 4). When CAL does not improve at early reassessment, endodontic versus periodontal attribution may remain indeterminate. Later observation may support classification as IC^+^_a_ or IC^+^_b_ based on apical healing, although IC^+^_b_ still reflects persistent uncertainty rather than definitive differentiation. (Figure 5).

#### 3.5.2. Grade b (IC^+^_b_): Uncertain Healing Tendency of Apical Periodontitis

In IC^+^ cases showing no reduction in CAL and no radiographic or CBCT evidence of apical healing following nonsurgical endodontic therapy, the healing tendency is considered uncertain. When this condition persists at later reassessment, the findings may reflect refractory apical periodontitis, marginal periodontitis, or a combined endodontic–periodontal pathology. In such situations, the pulpal–periodontal relationship cannot be clearly attributed to a single source, and clinical interpretation may need to consider both components rather than relying on the classification alone.

Within this framework, IC^+^_b_ represents alternative interpretive scenarios regarding the relative contributions of endodontic and periodontal factors (Figure 5):

- Sequential endo–perio interpretation.

Within this interpretive scenario, the endodontic component is considered first, followed by an interval of healing during which the periodontal condition is reassessed. When periodontal findings persist, the lesion may be interpreted as having a continuing periodontal component. Because this framework allows evaluation of periodontal changes after resolution of endodontic influence, periodontal findings may remain difficult to interpret at the time of early observation and can be reinterpreted after later observation, when lesion morphology becomes more distinguishable.

In some cases, this pattern of interpretation may correspond clinically to a staged surgical approach.

However, when features strongly suggest a persistent endodontic source—such as a lesion centered at the apex with a narrow isolated periodontal pocket, a persistent gingival abscess, or radiographic findings including extruded filling materials, fractured instruments, or ledges—the endodontic contribution may be interpreted as ongoing without requiring prolonged observation.

- Combined endo–perio interpretation.

In this interpretive scenario, endodontic and periodontal contributions are considered together, as the relative source of the lesion cannot be clearly separated. Clinical findings are therefore interpreted without assuming resolution of one component prior to assessment of the other. Consequently, periodontal findings may be evaluated without requiring a delay for resolution of the endodontic component.

In some cases, this pattern of interpretation may correspond clinically to a simultaneous surgical approach, in which both components are addressed within the same therapeutic episode.

This subdivision is intended to assist longitudinal clinical interpretation of treatment response rather than prescribe a specific surgical approach or to predict the histologic status of periodontal tissues.

## 4. Discussion

### 4.1. Significance of the New Framework

The proposed framework provides a structured approach to support clinical interpretation in the management of EPLs, addressing several limitations of previously proposed classification systems. Rather than focusing solely on etiologic categorization or the extent of periodontal destruction, this framework integrates two fundamental clinical considerations—pulp vitality and the presence or absence of IC—and relates them to patterns of clinical course and reassessment. Based on these two parameters, the framework discusses three clinical aspects: (i) considerations regarding pulp preservation in relation to the extent and location of viable pulp tissue; (ii) considerations for the timing of subgingival instrumentation and periodontal intervention, based on the clinical response following endodontic infection control; and (iii) interpretation of clinical situations in which surgical intervention may be encountered when nonsurgical management fails.

By linking diagnostic findings to treatment sequencing considerations, this framework aims to help clinicians interpret complex presentations of EPLs without relying solely on fixed treatment sequences. It offers a conceptual perspective that acknowledges the heterogeneous nature of EPLs and may assist in individualized case assessment. Accordingly, within this framework, IC should be interpreted as a clinical interpretive label that organizes reassessment and clinical monitoring, rather than as a diagnosis that determines treatment.

The following sections discuss clinical management patterns only as illustrative examples of how the interpretive framework may correspond to observed treatment decisions, rather than as prescriptive therapeutic recommendations.

### 4.2. Interpretation of Progression Patterns and Subclassification in V/IC^+^ and V/IC^−^ Cases

#### 4.2.1. Stepwise Assessment of Pulp Status in EPLs

EPT is adopted as an initial clinical reference in this framework because of its high sensitivity for detecting residual vital pulp, providing an initial indication of pulpal status. To support interpretation of clinical findings, EPT findings are complemented by cold testing and, when indicated, direct microscopic evaluation of the pulp chamber. This stepwise consideration allows for differentiation between mere pulpal vitality and the presence of viable pulp tissue suitable for preservation, thereby helping to recognize situations in which partial or complete pulp preservation may be clinically feasible.

The V/NV categorization derived from sensibility testing should not be interpreted as a definitive biological diagnosis of pulpal condition. Pulpal pathology exists along a biological continuum and frequently demonstrates heterogeneous patterns, particularly in multirooted teeth. Accordingly, the EPT-based assessment functions only as a provisional clinical categorization intended to structure observation and reassessment during management rather than establish a treatment-determining diagnostic algorithm. When vitality is suspected, further assessment may be considered, particularly in multirooted teeth in which heterogeneous pulpal conditions are suspected and full pulp preservation (V_1_) cannot be assumed. In such situations, root-specific evaluation and, when clinically indicated, intraoperative microscopic observation may be used to clarify the distribution of remaining vital tissue. In cases consistent with V_1_, management may proceed without pulpal intervention, accompanied by careful clinical monitoring and reassessment.

#### 4.2.2. Progression Patterns and Subclassification of V_2_ and V_3_

Grades V_2_ and V_3_ are applied to both V/IC^+^ and V/IC^−^ groups to describe the distribution of remaining viable pulp tissue, particularly in multirooted teeth; however, the underlying patterns of pulpal infection differ fundamentally between the two. In V/IC^+^ cases, bacterial invasion typically progresses in a coronoapical direction. When viable pulp tissue remains at the canal orifice level in at least one root, the lesion is classified as V_2_ and may correspond clinically to root-specific pulpotomy. If no such tissue is identified, the case is designated as V_3_, and clinical management often corresponds to complete RCT. By contrast, V/IC^−^ cases are generally associated with retrograde involvement of the root canal system originating from the apical foramen. In these cases, pulpal necrosis does not typically progress in a gradual apicocoronal manner. Instead, vascular compromise of the apical neurovascular bundle may lead to sudden and complete necrosis of the pulp within the affected root, while pulp tissue supplied by independent vascular pathways in other roots may remain vital [47]. In such situations, the condition corresponds to V_2_ when vitality is present in at least one root. With further progression, however, pulpal necrosis may extend beyond the canal orifice level in roots that were previously viable, ultimately leading to a clinical situation corresponding to V_3_. The framework does not exclude the theoretical possibility that limited vital tissue may persist in the apical third. However, because reliable clinical methods to confirm sterility and long-term functional preservation are currently lacking, the V_3_ category is intended to describe a predictable clinical pattern rather than mandate a specific therapeutic procedure.

#### 4.2.3. Diagnostic Challenges and Clinical Significance of V_1_ in V/IC^−^ Cases

The clinical interpretation process for borderline V_1_ cases is summarized as a stepwise interpretive scheme in Figure 2. In contrast to grades V_2_ and V_3_, which are differentiated following endodontic access, grade V_1_ is unique to V/IC^−^ cases and must be interpreted without pulpal intervention. Consequently, V_1_ represents the most diagnostically challenging grade within this framework, particularly in multirooted teeth. Accurate identification of V_1_ requires confirmation of clinically healthy, vital pulp without IC based on an integrated assessment of pulpal responsiveness and lesion progression rather than reliance on a single diagnostic test.

EPT alone is insufficient for diagnosing V_1_, as a positive response may be elicited as long as vitality is preserved in at least one root. This limitation underscores the need for a stepwise clinical interpretation incorporating additional clinical parameters. Importantly, radiographic radiolucency extending toward or around the apex does not necessarily indicate bacterial invasion of the pulp. A previous report has suggested that periodontal lesions may be spatially stratified and that more apically located regions may contain areas with reduced plaque accumulation and residual attachment. These findings indicate that the deepest region is not necessarily infected in all cases [48]. Therefore, radiographic findings must be interpreted cautiously in the context of EPLs.

Assessment of pulpal inflammation and infection risk may be further informed by cold testing and CAL evaluation. Cold testing offers a high negative predictive value for pulp vitality, such that a normal response strongly supports the presence of truly vital pulp tissue and suggests the absence of advanced pulpal inflammation [49]. In parallel, CAL that does not extend to the apex indicates that bacterial penetration into the pulp is unlikely. In such situations, the presence of remaining periodontal attachment adjacent to the apical foramen supports the interpretation that the pulp has not been infected, even when radiographic radiolucency appears extensive. Additionally, periodontal instrumentation may be performed with care to avoid extension into the apical foramen region to minimize potential injury to the apical neurovascular supply. This description is presented as a procedural consideration rather than a fixed distance-based criterion.

**Figure 6:**
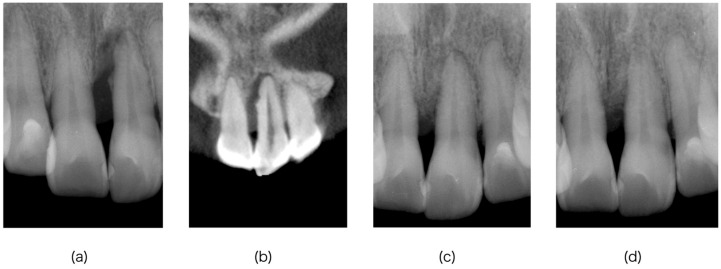
Representative clinical case demonstrating application of the proposed framework. (**a**) Initial periapical radiograph showing marginal bone loss extending toward the apical region. Clinical examination revealed a localized deep periodontal pocket with calculus accumulation, while the crown was intact without caries or defective restoration. The tooth responded normally to cold testing and electric pulp testing (EPT), with no spontaneous pain or percussion tenderness. (**b**) Cone-beam computed tomography imaging demonstrating that the radiolucency, although extending close to the apex, was not centered on the root apex and showed no features consistent with endodontic pathology. No sinus tract or coronal leakage was detected, and the lesion was interpreted as periodontal in origin (V_1_/IC^−^). (**c**) Three-month reevaluation after periodontal therapy showing clinical improvement without signs suggestive of pulpal pathology. (**d**) One-year follow-up radiograph demonstrating periodontal healing without development of periapical pathology. The tooth remained responsive to cold testing and EPT, confirming maintained pulp vitality.

Taken together, these findings justify a conservative interpretive approach in which V_1_ is provisionally identified and clinical monitoring may continue without endodontic intervention, provided that careful clinical follow-up is ensured. In clinical practice, differentiation between V_1_ and V_2_ may be challenging when attachment loss approaches the apical third despite normal pulp sensibility. In such situations, interpretation should not rely solely on the distance between the periodontal defect and apical foramen. Instead, the framework allows an initial observation phase to be considered when (1) cold testing indicates normal pulp response, (2) no clinical signs of pulpal infection are present, and (3) radiographic findings do not suggest endodontic involvement. Endodontic intervention may be reconsidered if healing is not observed during follow-up or if signs of pulpal pathology subsequently appear. This approach reflects the biological possibility that the pulp may remain uninfected despite advanced periodontal attachment loss. This observation phase does not represent passive monitoring; it includes active periodontal therapy and scheduled reevaluation to detect early pulpal breakdown. A representative clinical case demonstrating the clinical application of this interpretive process is shown in Figure 6. The case illustrates the clinical reasoning leading to a V_1_/IC^−^ diagnosis and its subsequent management. Accordingly, this case is presented to illustrate clinical application of the interpretive process and does not represent outcome validation of the framework.

Despite the use of multimodal diagnostic tools, accurate assessment of pulpal status remains challenging. Cold testing may elicit a positive response even in cases of partial necrosis, and variations in root anatomy and canal configuration further complicate interpretation. Moreover, even when the pulp is clinically considered vital, histological changes, such as inflammation or calcification, may be present [50,51]. Consequently, definitive confirmation of completely healthy pulp is often not achievable in routine clinical practice. Nevertheless, interpreting a case as grade V_1_ carries substantial clinical value, as it permits consideration of pulp preservation despite diagnostic uncertainty and allows clinicians to cautiously maintain the native pulp when the overall clinical context appears compatible with a non-infected pulp. Therefore, if the lesion does not resolve following periodontal therapy or if signs of secondary pulpal necrosis emerge, endodontic treatment may be reconsidered [52,53]. We acknowledge that long-term outcome data for V_1_ cases remain limited. Therefore, the interpretive approach should be interpreted as a biologically based clinical interpretation framework rather than a definitive prognostic prediction, and prospective clinical validation is necessary. Accordingly, V_1_ should be regarded as an interpretive condition that supports cautious monitoring rather than confirmation of pulpal health. In this category, endodontic treatment may be deferred in clinical practice while periodontal therapy and close reevaluation are undertaken. Ongoing advancements in high-resolution imaging, particularly magnetic resonance imaging (MRI), are expected to enhance the diagnostic precision and support the broader clinical use of this classification system [54].

### 4.3. Significance of IC in Interpreting the Treatment Interval

A treatment interval between endodontic and periodontal therapies has traditionally been recommended in EPLs to allow evaluation of periodontal changes following adequate control of endodontic infection. However, randomized clinical trials that directly compared treatment outcomes with and without such intervals found no significant advantages over delayed periodontal intervention [10,11,12], and some studies have even demonstrated superior periodontal healing when periodontal therapy was initiated immediately after endodontic treatment (Table 2).

Importantly, these randomized clinical trials predominantly included cases of periodontal pockets caused by marginal periodontitis, with minimal or no contribution from endodontic pathology. For example, in a study by Tewari et al., although the structural condition of the teeth was not fully described, there were no coronal defects in representative cases, suggesting that pulpal necrosis occurred secondary to marginal periodontitis rather than being attributable to IC. Therefore, these findings suggest that lesions primarily associated with periodontitis may show limited benefit from delaying periodontal intervention, and the value of a treatment interval may depend on the underlying disease process.

By contrast, in the presence of IC, endodontic pathology is more likely to contribute, at least in part, to periodontal pocket formation, although periodontal factors may also coexist. In such cases, resolution of intracanal infection through endodontic therapy may be followed by improvement of periodontal conditions, supporting subsequent clinical reassessment before periodontal intervention. This biological potential provides a clear rationale for establishing a treatment interval after endodontic therapy before initiating subgingival instrumentation. Within this framework, the presence of IC may, therefore, adopted as the primary criterion for determining whether a treatment interval is indicated. IC serves as a clinically practical consideration for interpreting possible endodontic contribution to lesion development and for helping clinicians interpret treatment sequencing in individual cases. From this perspective, lesions traditionally classified as primary endodontic lesions with secondary periodontal involvement and true combined lesions, as defined in Simon’s classification, can be collectively categorized as endodontic-contributing lesions (IC^+^). Although these entities have historically been considered difficult to distinguish diagnostically, their similar biological behavior associated with endodontic contribution supports a shared clinical interpretation of these presentations.

Within this framework, IC^+^ is interpreted as a clinical finding suggestive of a possible endodontic contribution, but it does not indicate the condition or extent of periodontal tissue destruction. Clinical presentation varies considerably among IC^+^ cases, and the severity of periodontal involvement cannot be reliably determined at initial diagnosis. Therefore, clinical response following initial endodontic infection control provides important information for subsequent clinical interpretation. Longitudinal reevaluation, including monitoring changes in the CAL, helps determine whether periodontal pockets resolve following endodontic infection control or persist as true periodontal defects requiring periodontal therapy. Accordingly, an observation and reassessment period (often approximately 3 months in clinical practice) may be considered before considering subgingival instrumentation. This interval allows for biologically meaningful reassessment while supporting interpretation of treatment sequencing and minimizing unnecessary therapeutic invasiveness. Importantly, this interval is not intended as a fixed treatment directive but as a practical period for clinical reassessment, and earlier periodontal intervention may be appropriate when periodontal progression is evident.

Conversely, an initial IC^−^ interpretation does not eliminate the possibility of endodontic involvement. False-negative assessment may occur, particularly in vital multirooted teeth with preserved overall pulp sensibility despite possible root-specific necrosis, or in previously treated teeth with uncertain intraradicular status. For this reason, the framework incorporates structured follow-up monitoring after initial management. If periodontal healing is not achieved or signs of pulpal or endodontic pathology appear during follow-up, endodontic treatment may then be considered based on the evolving clinical findings. Accordingly, clinical interpretation should incorporate follow-up evaluation when uncertainty remains.

### 4.4. Clinical Interpretation of Treatment Response in IC^+^_b_ Cases

While the general principles of surgical decision-making in EPLs were outlined in Section 2.3.3, this section specifically addresses the interpretive application of those principles in IC^+^_b_ cases—where both apical and marginal pathologies may coexist and etiologic attribution may remain uncertain during reassessment.

When nonsurgical therapy fails to achieve resolution, interpretation of persistent findings and the clinical contexts in which surgical intervention may be considered have not been adequately systematized in previous classification frameworks. Two key clinical challenges underlie this lack of systematization.

First, the timing of lesion re-evaluation using CBCT or other imaging modalities has not been standardized. Even when nonsurgical endodontic treatment is successful, complete radiographic resolution of the lesion may require up to four years [55]. Therefore, confirming the elimination of endodontic inflammation based solely on complete lesion disappearance is impractical in EPLs. Instead, an interval of approximately 6–12 months is often used to interpret the healing tendency. Lesions that exhibit radiographic improvement within this period are considered likely to continue healing thereafter [22,46], so findings during this timeframe may provide useful context for interpreting the response to nonsurgical endodontic therapy.

Second, in cases classified as IC^+^_b_—where no improvement in CAL and no radiographic or CBCT evidence of apical healing are observed—it is often difficult to identify the primary etiology. These lesions may involve refractory apical periodontitis, marginal periodontitis, or a combination of both.

In this context, clinical findings are often interpreted in relation to two possible clinical contexts: a "staged surgical approach" or "simultaneous surgical approach". These approaches are discussed here as clinical contexts that may help interpret the relative endodontic and periodontal contributions, rather than as prescriptive treatment recommendations.

The staged approach may be interpreted as consistent with situations in which refractory apical periodontitis is suspected, as it can avoid unnecessary regenerative therapy [36,37,38]. However, if marginal periodontitis contributes to the lesion, this approach may prolong treatment, require multiple surgical sessions, and result in gingival recession after the first surgery, potentially complicating the regenerative phase. Moreover, delaying subgingival instrumentation in such cases may allow further progression of marginal periodontitis. Therefore, a staged approach may reflect cases in which the marginal periodontal component is limited based on comprehensive clinical and radiographic findings. Additionally, if significant root surface contamination (e.g., calculus) is observed intraoperatively during endodontic surgery, regenerative therapy may also be performed in the same session depending on intraoperative findings.

The simultaneous approach represents a clinical situation in which both endodontic and periodontal components are addressed concurrently and may be encountered in situations where marginal periodontitis is suspected. It permits earlier subgingival instrumentation, reflecting consideration of a potential periodontal component. This strategy also offers a shorter treatment timeline and requires fewer surgical sessions, which may appeal to both clinicians and patients. However, this requires better surgical skills and incurs higher procedural costs. Notably, many studies reporting high success rates for this approach have involved apicomarginal lesions treated without prior nonsurgical endodontic therapy [40,41,42,43], raising concerns that outcomes in IC^+^_b_ cases may not mirror those reported in the literature. Further research is required to clarify its prognostic reliability in this specific context.

No definitive evidence currently favors one clinical context over the other in IC^+^_b_ cases. Instead, the observed clinical course should be interpreted in light of multiple factors, including:

- Etiologic risk estimation (apical vs. marginal involvement);
- Presence of intraoperative findings (e.g., calculus);
- Likelihood of periodontal improvement after endodontic management;
- Overall periodontal status;
- Patient-specific factors (preference, systemic conditions, compliance, etc.);
- Technical feasibility and cost.

These factors do not determine treatment selection but may assist in interpreting the relative endodontic and periodontal contributions. Clinical findings observed during surgical management in IC^+^_b_ may therefore provide additional information that supports reassessment of the pulpal–periodontal condition over time. Accordingly, the concepts presented in this framework are derived primarily from biological rationale, clinical observations, and synthesis of existing literature rather than from direct outcome-based clinical trials. The framework should therefore be understood as an integrative conceptual model intended to organize clinical interpretation and generate testable hypotheses, rather than as evidence-based treatment guidance.

### 4.5. Limitations and Future Perspectives in Clinical Application

The proposed classification system is intended to provide a structured framework for organizing clinical interpretation of EPLs. However, several limitations should be acknowledged. First, both pulp vitality testing and assessment of IC are susceptible to false-negative and false-positive findings, and their diagnostic accuracy remains operator-dependent, often requiring adjunctive diagnostic aids. Second, the proposed interval between endodontic and periodontal therapies and the timing of surgical intervention are supported primarily by indirect or retrospective evidence. Prospective, well-controlled clinical studies are therefore necessary to establish reproducibility and external validity.

In addition, from a clinical implementation perspective, application of the framework may depend on clinician experience, careful examination under magnification, and interpretation of radiographic or CBCT findings. Because the strategy incorporates a period of observation and reassessment prior to certain interventions, treatment duration may be longer than conventional immediate treatment approaches, which may limit its use in patients with poor compliance or unreliable follow-up. Accordingly, careful case selection and appropriate patient communication are important when applying the framework.

Prospective multicenter validation is necessary before broad clinical adoption of the proposed framework. Further studies with standardized protocols and long-term outcome assessment are warranted to determine its generalizability and reproducibility.

Accordingly, the proposed framework should be regarded as a hypothesis-generating conceptual model intended to support clinical reasoning rather than to replace existing diagnostic or therapeutic approaches. It should be applied in conjunction with comprehensive clinical evaluation and clinician judgment and should not be used in isolation for treatment decision-making.

## 5. Conclusions

This review presents a conceptual interpretive framework for interpreting EPLs based on two clinical considerations: (i) pulp vitality and (ii) the presence or absence of IC. These considerations are used to describe four primary clinical groups, with additional grading according to the extent of remaining viable pulp and persistence of possible etiologic factors following initial management. By integrating these considerations, the proposed framework is intended to assist structured clinical interpretation and reassessment rather than to determine definitive treatment strategies.

Rather than replacing existing treatment approaches, the V/IC framework offers a structured method for organizing clinical findings and facilitating communication regarding the heterogeneous presentations of EPLs. Prospective validation and assessment of reproducibility are warranted before routine clinical adoption.

Although prospective clinical validation and long-term follow-up are required before routine clinical application, the proposed approach may provide a basis for more consistent case assessment and discussion of EPLs presentations.

## Figures and Tables

**Table 1:** Grades for pulp preservation (V group).

Grade	Applicable IC Status	Diagnostic Condition	Clinical Interpretation
V_1_	IC^−^ only	CAL does not reach the apex; normal response to cold testing; debridement can be performed without contacting the apex; absence of IC	Pulp is clinically healthy and vital, with no clinical signs suggestive of pulpal infection, indicating favorable conditions for full pulp preservation without endodontic intervention
V_2_	IC^−^/IC^+^	Coronal pulp shows infection, while vital pulp tissue remains at the canal orifices in one or more roots and is considered viable under microscopic observation in a multirooted tooth	Pulp vitality is heterogeneous among roots, allowing for partial preservation of viable pulp tissue, while infection control is required in other roots
V_3_	IC^−^/IC^+^	No viable pulp tissue observed at the canal orifice level in any root	Residual vital pulp tissue is presumed to be confined to the apical third and is insufficient for pulp preservation

This grade applies to vital-pulp cases (V/IC^+^ and V/IC^−^). Diagnostic assessment is primarily based on electric pulp testing (EPT), supplemented by microscopic examination and cold testing. CAL = clinical attachment level; IC = coronal-originating infection.

**Table 2:** Summary of randomized clinical trials comparing outcomes with and without intervals between endodontic and periodontal therapy.

Author	Lesion Type	Pulpal Status	Tooth Structure Loss	Apical/Alveolar Involvement	Pocket Characteristics	Communication
Gupta et al. [10]	Concurrent endo–perio lesion without communication	Non-vital, untreated pulp	Not specified	Presence of apical radiolucency	PD ≥ 5 mm (wide-based pocket)	Absent
Dong et al. [11]	Severe combined lesion (vital or slightly reduced pulp sensibility)	Vital to slightly reduced	No tooth structure loss	Vertical alveolar bone resorption extending to the apical third of the root	Deep pocket (PD > 6 mm, CAL > 4 mm)	Not explicitly stated
Tewari et al. [12]	Concurrent endo–perio lesion with apical communication	Untreated, non-vital pulp	Not specified	Presence of apical radiolucency	Wide periodontal pocket extending to the apex	Present

All trials compared periodontal healing with and without intervals following root canal treatment. Tewari et al. and Dong et al. addressed true combined endo–periodontal lesions with communication, whereas Gupta et al. included marginally derived lesions without endo communication. PD = pocket depth; CAL = clinical attachment level.

## Data Availability

No new data were created or analyzed in this study. Data sharing is not applicable to this article.

## Abbreviations

The following abbreviations are used in this manuscript:

AAE	American Association of Endodontists
AAP	American Academy of Periodontology
CAL	Clinical attachment level
CBCT	Cone-beam computed tomography
CEJ	Cementoenamel junction
EFP	European Federation of Periodontology
EPLs	Endodontic–periodontal lesions
EPT	Electric pulp testing
ESE	European Society of Endodontology
IC	Coronal-originating infection
IC^+^	Presence of coronal-originating infection
IC^+^_a,b_	Grades of apical healing after endodontic treatment
MRI	Magnetic resonance imaging
NV	Non-vital (pulp)
RCT	Root canal treatment
V	Vital (pulp)
V_1_–V_3_	Grades of the extent of remaining viable pulp
